# Improving Access to Healthcare in Sierra Leone: The Role of the Newly Developed National Emergency Medical Service

**DOI:** 10.3390/ijerph18189546

**Published:** 2021-09-10

**Authors:** Marta Caviglia, Marcelo Dell’Aringa, Giovanni Putoto, Riccardo Buson, Sara Pini, Daniel Youkee, Amara Jambai, Matthew Jusu Vandy, Paolo Rosi, Ives Hubloue, Francesco Della Corte, Luca Ragazzoni, Francesco Barone-Adesi

**Affiliations:** 1 CRIMEDIM—Center for Research and Training in Disaster Medicine, Humanitarian Aid, and Global Health, Università del Piemonte Orientale, 28100 Novara, Italy; marcelo.dellaringa@uniupo.it (M.D.); francesco.dellacorte@med.uniupo.it (F.D.C.); luca.ragazzoni@med.uniupo.it (L.R.); francesco.baroneadesi@uniupo.it (F.B.-A.); 2 Research Section, Doctors with Africa CUAMM, 35121 Padua, Italy; g.putoto@cuamm.org (G.P.); r.buson@cuamm.org (R.B.); sarapini89@gmail.com (S.P.); 3 School of Population Health and Environmental Sciences, King’s College London, London SE5 9NU, UK; daniel.youkee@kcl.ac.uk; 4 Ministry of Health and Sanitation, Government of Sierra Leone, Freetown, Sierra Leone; amarajambai@yahoo.com (A.J.); matthewjusuvandy@yahoo.co.uk (M.J.V.); 5 SUEM 118 Venezia, Azienda ULSS 3 Serenissima, 30174 Mestre, Italy; paolo.rosi@aulss3.veneto.it; 6 Research Group on Emergency and Disaster Medicine, Vrije Universiteit Brussels, 1050 Brussels, Belgium; Ives.Hubloue@vub.be

**Keywords:** Free Health Care Initiative (FHCI), National Emergency Medical Service (NEMS), emergency medical service (EMS), primary health units (PHUs), operation center (OC)

## Abstract

We aim to evaluate whether the first National Emergency Medical Service (NEMS) improved access to hospital care for the people of Sierra Leone. We performed an interrupted time-series analysis to assess the effects of NEMS implementation on hospital admissions in 25 facilities. The analysis was also replicated separately for the area of Freetown and the rest of the country. The study population was stratified by the main Free Health Care Initiative (FHCI) categories of pregnant women, children under 5 years of age, and populations excluded from the FHCI. Finally, we calculated direct costs of the service. We report a 43% overall increase in hospital admissions immediately after NEMS inception (RR 1.43; 95% CI 1.2–1.61). Analyses stratified by FHCI categories showed a significant increase among pregnant women (RR 1.54; 95% CI 1.33–1.77) and among individuals excluded from the FHCI (RR 2.95; 95% CI 2.47–3.53). The observed effect was mainly due to the impact of NEMS on the rural districts. The estimated recurrent cost per ambulance ride and NEMS yearly cost per inhabitant were 124 and 0.45 USD, respectively. To our knowledge, this is the first nationwide study documenting the increase in access to healthcare services following the implementation of an ambulance-based medical service in a low-income country. Based on our results, NEMS was able to overcome the existing barriers of geographical accessibility and transport availability, especially in the rural areas of Sierra Leone.

## 1. Introduction

Multiple barriers prevent an equitable access to healthcare services in low-income countries (LICs), leading to higher morbidity and mortality rates for both acute and chronic diseases, especially in rural communities [1,2,3].

Sierra Leone is one of the least developed countries worldwide, where access to healthcare is mostly constrained by geographical barriers, extremely high out-of-pocket expenditures, lack of skilled medical staff, and poor service quality [4,5]. Furthermore, the country’s health resources are unevenly distributed, with the vast majority of referral hospitals and more than half of the entire workforce concentrated in the urban area of Freetown, the nation’s capital [5].

To tackle the high rates of maternal and neonatal mortality reported in its territory, in 2010, the Sierra Leone government launched the Free Health Care Initiative (FHCI), which waived all medical-related fees for pregnant and breastfeeding women, children under the age of 5 years, and Ebola survivors [6]. Although the FHCI was effective in curbing financial barriers to accessing care, leading to an overall improvement in the utilization rates of healthcare services, wealth-related health inequalities remained prevalent, and people residing in rural areas, accounting for 59% of the total population, were still left underserved [7,8].

Similar to most of the other African countries, Sierra Leone has been long devoid of any formalized prehospital care system [9]. Since the limited number of ambulances available in the country were associated with high transport-related fees, the majority of patients used to reach hospital facilities either using private vehicles or public services, therefore bearing the costs of transport fare and often being subjected to delays in care [10]. 

In line with recommendations issued by the World Health Organization (WHO), in 2013, the African Federation of Emergency Medicine advocated for the development of prehospital care systems to reduce the high morbidity and mortality rates reported in African countries [11,12]. Thus, in keeping with both WHO guidelines and national health security policies, in 2016, a government-backed joint venture comprising Doctors with Africa (CUAMM, Padua, Italy), the Regional Government of Veneto (Italy), the Research Center in Emergency and Disaster Medicine (CRIMEDIM, Università del Piemonte Orientale, Italy), and the Sierra Leone Ministry of Health and Sanitation (MOHS), designed the first National Emergency Medical Service (NEMS) in Sierra Leone, one of the very few coordinated, structured, and fully equipped prehospital emergency medical services (EMS) in the African continent [5,13,14]. The goal of this newly developed entity is to provide a free-of-charge prehospital service coordinated by a centralized operation center (OC), using part of the ambulances donated to the country during the Ebola outbreak [14]. With seed funding from the World Bank (Washington, DC USA), the service started in October 2018 and reached the full operativity countrywide on the 27th of May 2019, after a gradual process of training sessions and subsequent activation in all the 14 districts of Sierra Leone [14]. After a 26-month work plan, the joint venture released a fully staffed and functional NEMS working all over Sierra Leone, managed by the local MOHS staff and funded through the governmental budget.

The aim of the present study was to evaluate the impact of this initiative on access to hospital care for the general population, with a special emphasis on underserved rural communities. We also provided an overview of the direct costs of the service. 

## 2. Materials and Methods

### 2.1. Study Setting and Design

**Figure 1:**
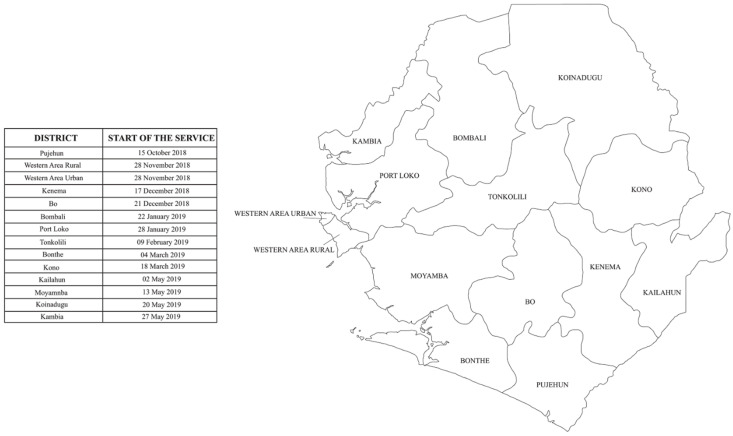
Time of implementation of the National Emergency Medical Service (NEMS) in the 14 districts of Sierra Leone.

This was a retrospective study using monthly aggregated data on hospital admissions across Sierra Leone, recorded between October 2017 and November 2019. The study encompassed 25 hospital facilities distributed across the 14 districts of Sierra Leone, including government district hospitals, faith-based clinics, and health centers managed by non-governmental organizations. As the NEMS was introduced in the various districts at different times (from the 15 October 2018 to the 27 May 2019; Figure 1), we analyzed data collected 12 months before and 6 months after NEMS inception for each district.

The NEMS ambulance referral system has been designed as a tiered system of care, comprising peripheral health units (PHUs), in charge of the primary assessment and care of patients, a fleet of 81 ambulance units, and healthcare facilities at different levels [14]. It recognizes two main categories of interventions: “red” codes, which are clinically defined as “immediately life threatening”, and “yellow” codes, which are clinically defined as “not life threatening but still serious.” At the time of this research, NEMS activation was initiated by a registered nurse, midwife, or healthcare worker at the PHU level, and all referred cases were appropriately evaluated and managed by trained nurses allocated to the NEMS OC through codes and scripted questions adapted from the Medical Priority Dispatch system [15], available on request from the authors. Triage accuracy and adherence to protocols were overseen by the OC Supervisor [14], and a post-hoc cross-check was performed in the category of pregnant women to confirm that the severity of the diagnosis reported coincided with the triage category assigned. 

Ambulances were staffed by a paramedic and an ambulance care driver, trained in the basic principles of prehospital emergency care, including management of medical emergencies, trauma, obstetrics and gynecologic and pediatric emergencies [14]. Treatments performed in the ambulances included oxygen delivery, continuation of fluid administration after peripheral intravenous cannulation performed by nurses at the PHU level, assistance during labor and delivery, administration of rectal misoprostol for the prevention of postpartum hemorrhage, and basic life support and resuscitation maneuvers without the support of automated external defibrillator. 

### 2.2. Data Collection

Data were retrieved from the national referral coordinators’ database, storing information on all incoming referrals collected at each hospital facility [16]. After initial notification of the incoming referral via telephone call from the sending PHU, patients were identified upon arrival by referral coordinators, and information regarding patient demographics, clinical condition, mode, and time of transport to the referral facility were collected through a paper report form, which was then transcribed into an Epi Info™ datasheet (Centers for Disease Control and Prevention, Atlanta, GA, USA). Data entry integrity and accuracy was monitored with monthly inspection by data collector supervisors. 

Referral and in-hospital data have been recorded nationwide by the network of local referral coordinators since September 2017, before the implementation of NEMS, when the vast majority of patients used either private or public means of transport to reach the hospital from the peripheral health units. This structured data collection system has been incorporated in the NEMS system upon its inception, and scans of patient referral forms filled by ambulance teams have been used to crosscheck data collected by referral coordinators at the hospital level. 

Data extracted for this research included age, gender, FHCI category, priority (“urgent” or “non-urgent”), date and time of arrival at the hospital, and transport mode (“NEMS ambulance” or “other”). 

Data on the population of Sierra Leone and its districts were extracted from the 2015 Sierra Leone Census, as reported on the Sierra Leone Statistics website [8]. Additional data on costs were retrieved from the NEMS monthly financial and management internal reports from the 1 June 2019 until the 31 March 2020, during which NEMS worked at full capacity. Data included the recurrent cost of personnel, the fuel for ambulance referral (based on kilometers covered as recorded through the global positioning system (GPS) and mean consumption per kilometer), and the fuel for the generator at the NEMS OC, maintenance, insurance, GPS, medical material and personal protective equipment, and mobile phone expenses.

### 2.3. Statistical Analysis

We adopted standard methods for interrupted time-series to assess the effects of the introduction of the NEMS [17,18]. We carried out negative binomial regression models to account for the possible overdispersion of data. In all considered models, the dependent variable was the monthly number of hospital admissions and the population size was included as the offset. Among the independent variables of the model, the immediate effect of the intervention was modeled as a step function, using an indicator variable and taking a value of 1 at the time of implementation of NEMS in each district, whereas the gradual effects were investigated considering an interaction term between the introduction of NEMS and time [17]. To explore the effect of the introduction of NEMS on the different FHCI categories, the study population was classified according to the following groups: pregnant women, children under 5 years of age, and populations without access to free healthcare. We excluded from the analysis the remaining FHCI categories of Ebola survivors and breastfeeding women, as they included a very small number of subjects (less than 5% of the total). We performed the main analysis for the whole country, but we also carried out separate analyses for the area of Freetown and the rest of the country. We performed the analysis using Stata15 ( StataCorp. 2017, College Station, TX, USA). All tests were two-sided and performed at the 5% level of statistical significance. 

Calculation of the cost included recurrent costs of the intervention, while capital costs have been excluded from the analysis as the implementation phase of the project was entirely funded by the World Bank and relied on vehicles donated from around the world during the 2014–2016 Ebola crisis.

## 3. Results

A total of 28,574 hospital admissions in the 14 districts of Sierra Leone were included in the analysis. Table 1 shows the demographic characteristics of the analyzed population. 

Both in the period before and after the implementation of the NEMS, women’s access to hospital facilities was much higher compared to men. The majority of hospital admissions were represented by patients included in the FHCI, namely pregnant women and children under 5 years of age (Table 1).

**Figure 2:**
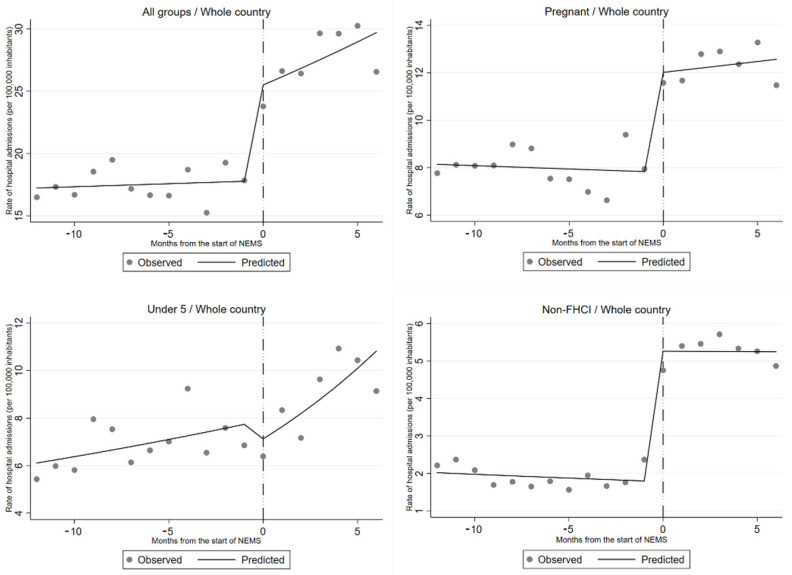
Observed and predicted hospital admission rates in Sierra Leone before and after the introduction of the NEMS. The results are also stratified by categories of the Free Health Care Initiative.

An overview of the obstetric diagnosis associated with “red” codes assigned in the category of pregnant women is presented in the supplementary Table S1. After NEMS inception, 80.1% of the patient arrived at hospitals using NEMS ambulances, and in 97.8% of the cases, the mission priority was deemed as “urgent” upon arrival at the health facilities. A 43% increase in hospital admissions was observed in Sierra Leone immediately after the introduction of the NEMS (RR 1.43; 95% CI 1.26–1.61) (Table 2 and Figure 2).

**Figure 3:**
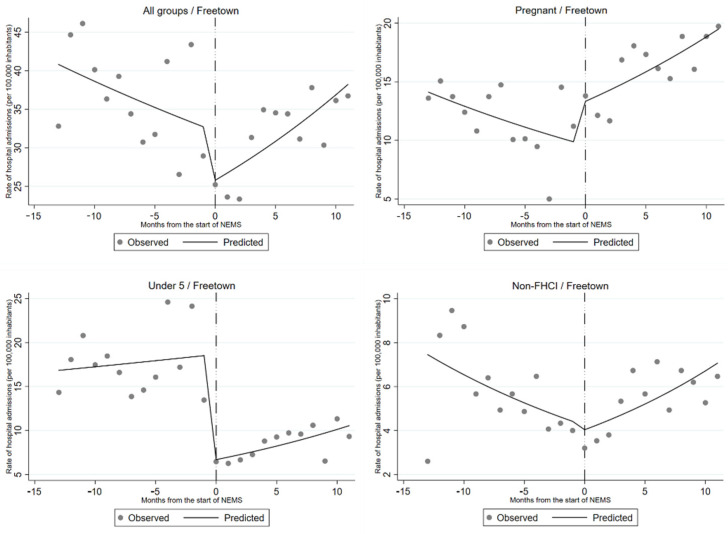
Observed and predicted hospital admission rates in Freetown before and after the introduction of the NEMS. The results are also stratified by categories of the Free Health Care.

In the different subgroups, a statistically significant increase was reported among pregnant women (RR 1.54; 95% CI 1.33–1.77) and those exempted from the FHCI (RR 2.95; 95% CI 2.47–3.53), but not for children under 5 years of age (RR 0.90; 95% CI 0.72–1.13). We did not find evidence of an additional gradual effect of the intervention over time, as there was no statistically significant change in the underlying trend in admissions after the implementation of the NEMS. Analyses stratified by area showed a very different pattern in Freetown compared to the rest of the country (Table 3). Overall, there was no increase in hospital admissions in Freetown. Pregnant women reported a 39% (RR 1.37; 95% CI 1.05–1.84) immediate increase and a monthly 6% (RR 1.06; 95% CI 1.02–1.11) gradual increase following the activation of the NEMS, while a substantial drop (RR 0.36 95% CI 0.27–0.47) in hospital admissions was observed among children under 5 years of age (Figure 3). Access to a hospital for subjects not included in the FHCI remained stable over time. 

**Figure 4:**
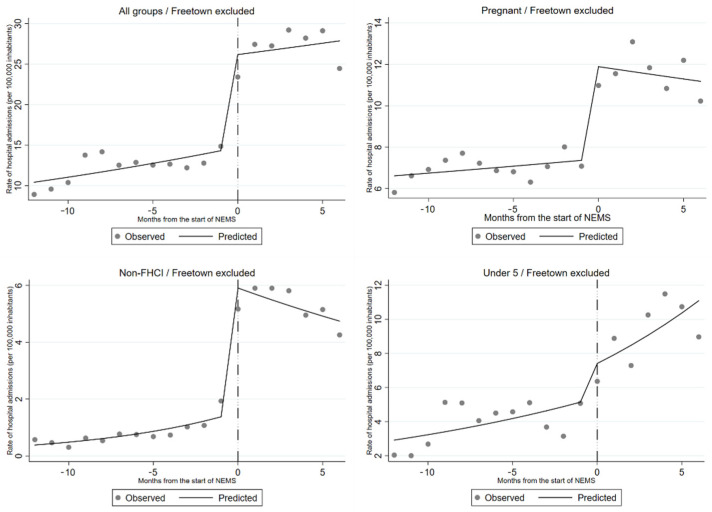
Observed and predicted hospital admission rates in Sierra Leone, with the exclusion of Freetown, before and after the introduction of NEMS. The results are also stratified by categories of the Free Health Care Initiative.

Conversely, in the rest of the country an immediate increase in the number of hospital admissions was evident both overall and for all considered subgroups, except for children under 5 years of age (Table 4 and Figure 4).

Table 5 shows the estimated costs of the intervention. The majority of expenses were composed of personnel costs. The estimated recurrent cost per ambulance ride was 124 USD, based on an average monthly number of 2366 NEMS missions. The NEMS’ yearly cost per inhabitant was 0.45 USD.

## 4. Discussion

The results of our study show that the implementation of NEMS in Sierra Leone was associated with an overall increase in access to hospitals. The rise in hospital admissions was observed not only among individuals with free access to healthcare services, but also among those not exempted from payment, suggesting that prior to NEMS inception, geographical and transportation barriers played an important role in limiting the access to the country’s healthcare services, irrespective of the possible financial constraints of subjects. This aspect corroborates findings from studies performed in other LICs, where distance or travel time to health facilities was inversely related to the healthcare seeking behavior of the population [19,20].

Moreover, the observation that underserved rural communities benefited the most from the activation of the free ambulance service in terms of ability to reach hospital care suggests that NEMS was effective in reducing health inequalities between urban and rural areas in Sierra Leone. Fittingly, we did not observe any major increment in hospital admission rates in the Freetown area, the most heavily populated district in the country, characterized by a higher concentration of healthcare facilities and better road conditions. Indeed, it is well established that urban residents of LICs have a much better prospect of accessing healthcare services compared to that of people living in rural communities, inevitably affected by structural constraints and health inequities [21,22,23].

The results stratified by FHCI categories show an increase in the number of hospital admissions of pregnant women upon NEMS inception. Although healthcare service utilization rates among pregnant women increased after the implementation of FHCI, this improvement was deemed not adequate enough to meet the sustainable development goals of the country [7,24,25]. Indeed, the FHCI had to face a number of issues hampering its effectiveness, first and foremost the non-affordability of transport-related indirect costs for people residing in rural areas [7,26,27] Moreover, the decision to seek care in health facilities during childbirth in rural Sierra Leone is also affected by a number of cultural and sociodemographic factors that often limit the impact of public health initiatives [26,28]. Although it is argued that the sole availability of EMS does not equal access to care when barriers related to the social acceptability of the initiatives are not fully addressed [29], our findings suggest that the development of a nationwide referral system (e.g., NEMS) may be a key strategy to boost the effects of other healthcare interventions already in place. Notwithstanding the countrywide support through awareness and public sensitization campaigns [14], future studies will be useful to assess the current acceptability of NEMS among the population of Sierra Leone and to inform public health initiatives.

Another interesting finding of our study is the decreased number of hospital admissions among “children under 5” in the Freetown area, which was evident immediately after the implementation of the NEMS. This observation may be explained by the presence of a big referral facility in Freetown, the Ola During Children’s Hospital, which used to treat a substantial number of pediatric cases from other districts before NEMS inception. Our results suggest that the NEMS allowed a better distribution of pediatric patients among the districts surrounding the capital, with a possible improvement in the use of available healthcare services.

To our knowledge, this is the first nationwide study documenting the impact of a newly developed EMS on health service utilization rates in a LIC. Although previous studies performed in low- and lower middle-income African countries attempted to evaluate the few existing referral systems, analyses were restricted to the local, district, or regional level [30,31,32,33,34,35].

A relevant aspect that arose from the implementation of NEMS in Sierra Leone was the importance of developing a robust and reliable patient referral network, featuring an efficient communication network and well-defined standard operating procedures [14]. Referral systems in LICs are often compromised by systemic inefficiencies, including a lack of coordination between the different healthcare facilities levels, which either cause a delay in access to care or lead to over congestion of referral hospitals [36]. In Ghana, for example, poor communication between referrers and receivers has been shown to undermine the entire referral process [33,37]. Additionally, despite being key to assess the status and the performance of the entire EMS system, monitoring and quality assurance programs are frequently either absent or only partially developed among African LICs [9,34]. Our results indicate that almost all patients transported to hospital facilities met the priority criteria identified for NEMS interventions, suggesting a high compliance with the NEMS standard operating procedures for referrals and effective communication at different levels. Furthermore, the integration of the national referral coordinators’ database into the NEMS information system, which allowed for continuous monitoring of NEMS operations at the national level, represented an additional point of strength of the project, allowing to understand the demand of emergency services and to adopt corrective and preventive measures. In this regard, we believe that processes of continuous oversight, supervisory actions, and monitoring efforts contributed greatly to achieve such a level of efficiency. 

Of note, the vast majority of hospital admissions recorded after the implementation of NEMS involved the use of NEMS ambulances. This high utilization rate is associated with an average cost of the service that is slightly higher compared to other African countries, where, however, referral systems are only regionally developed and less structured than NEMS [38,39]. It is also worth mentioning that a significant share of the total expenditure is related to wage expenses, as NEMS fleet accounts for 930 ground personnel, and the monthly remuneration of 250 USD offered to NEMS paramedics is higher than the average salary of nurses working in public hospitals, in order to attract and retain trained personnel [40].

The findings of this study should be interpreted considering some limitations. First, although the systematic collection of data was deemed accurate, information regarding admission and discharge diagnoses of patients was recorded as free text and were often either unreliable or missing. Additionally, information regarding in-hospital treatment was missing. For this reason, it was not possible to evaluate whether the implementation of the NEMS effectively translated in better clinical outcomes for patients nor if hospital admission had any detrimental effect on patients excluded from the FHCI who had to bear out of pocket expenditures to receive treatments. To overcome these technical limitations, we plan to perform additional studies on selected healthcare facilities, where more accurate clinical information might be available. Moreover, as the data analyzed did not include patients’ follow-up after hospital discharge, we were unable to provide information on how patients returned home from hospital facilities, and patients’ perspectives were not investigated in a qualitative manner. 

As in any observational study, the role of possible confounders cannot be definitely ruled out. However, confounding in time series studies is usually limited to factors that are related to the outcome of interest and change at the time of the intervention [41]. In contrast, temporal changes in the prevalence of individual risk factors shape the underlying long-term trends and are, thus, inherently considered in this kind of analysis. To our knowledge, with the exclusion of NEMS, no other nation-wide intervention potentially able to cause a sharp increase in rates of hospital admissions took place in Sierra Leone during the study period. Moreover, NEMS was implemented at different times in the different districts, over an 8-month timespan, and the effect appeared almost immediately after the intervention. These two facts make it unlikely that unmeasured confounding played a major role in the observed results.

## 5. Conclusions

In conclusion, the present study demonstrates that the implementation of NEMS in Sierra Leone enhanced access to hospital care among vulnerable populations by overcoming existing barriers, such as geographical accessibility and transport availability, especially in the rural areas of the country. Our findings suggest that NEMS was able to boost the effects of the FHCI, therefore building on the existing governmental strategy to improve maternal and child health indicators and to achieve the sustainable development goals of Sierra Leone. Altogether, our findings may serve as the basis for the development and evaluation of prehospital emergency and referral services in other LICs.

## Figures and Tables

**Table 1:** Demographic characteristics of admitted patients before and after the introduction of the National Emergency Medical System (NEMS).

	Pre NEMS (12-Months Timeframe)	Post NEMS (6-Months Timeframe)
**Gender, *n* (%)**		
Female	10,741 (72.1)	9805 (71.7)
Male	4150 (27.9)	3878 (28.3)
**FHCI category, *n* (%)**		
Pregnant	6799 (45.6)	6104 (44.6)
Lactating	533 (3.6)	573 (4.2)
Under 5	5865 (39.4)	4398 (32.1)
Ebola Survivors	71 (0.5)	0 (0)
Non-Free Healthcare	1623 (10.9)	2608 (19)
**Districts, *n* (%)**		
Bo	692 (4.6)	1129 (8.2)
Bombali	658 (4.4)	941 (6.9)
Bonthe	335 (2.2)	341 (2.5)
Kailahun	574 (3.8)	668 (4.9)
Kambia	574 (3.8)	678 (4.9)
Kenema	837 (5.6)	1000 (7.3)
Koinadugu	531 (3.6)	480 (3.5)
Kono	450 (3.0)	902 (6.6)
Moyamba	417 (2.8)	457 (3.3)
Port Loko	882 (5.9)	833 (6.0)
Pujehun	2019 (13.6)	1643 (12.0)
Tonkolili	270 (1.8)	1501 (10.9)
Western Area	6652 (44.7)	3100 (22.6)
Total, *n*	14,891	13,683

**Table 2:** Effect of the introduction of the NEMS on hospital admission rates in Sierra Leone. The results are presented as immediate and gradual (monthly) change in the rates of hospital admissions after the introduction of the intervention.

Group	Effect of the Intervention	Rate Ratio (RR)	95% CI	*p*-Value
All	Immediate	1.43	1.26 to 1.61	<0.001
	Gradual	1.02	0.99 to 1.05	0.103
Pregnant	Immediate	1.54	1.33 to 1.77	<0.001
	Gradual	1.01	0.98 to 1.04	0.505
Under 5	Immediate	0.90	0.72 to 1.13	0.362
	Gradual	1.05	0.99 to 1.10	0.062
Non-FHCI ^1^	Immediate	2.95	2.47 to 3.53	<0.001
	Gradual	1.01	0.97 to 1.05	0.603

^1^ Non-FHCI = not included in the Free Health Care Initiative.

**Table 3:** Effect of the introduction of the NEMS on hospital admission rates in Freetown. The results are presented as immediate and gradual (monthly) change in the rates of hospital admissions after the introduction of the intervention.

Group	Effect of the Intervention	Rate Ratio (RR)	95% CI	*p*-Value
All	Immediate	0.80	0.65 to 0.99	0.041
	Gradual	1.05	1.02 to 1.09	<0.001
Pregnant	Immediate	1.39	1.05 to 1.84	0.021
	Gradual	1.06	1.02 to 1.11	0.001
Under 5	Immediate	0.36	0.27 to 0.47	<0.001
	Gradual	1.03	0.99 to 1.07	0.090
Non-FHCI	Immediate	0.95	0.63 to 1.44	0.825
	Gradual	1.09	1.04 to 1.16	0.001

Non-FHCI = not included in the Free Health Care Initiative.

**Table 4:** Effect of the introduction of NEMS on hospital admission rates in Freetown. The results are presented as immediate and gradual (monthly) change in the rates of hospital admissions after the introduction of the intervention.

Group	Effect of the Intervention	Rate Ratio (RR)	95% CI	*p*-Value
All	Immediate	1.77	1.49 to 2.11	<0.001
	Gradual	0.98	0.94 to 1.02	0.358
Pregnant	Immediate	1.59	1.40 to 1.82	<0.001
	Gradual	0.98	0.95 to 1.00	0.179
Under 5	Immediate	1.36	0.88 to 2.11	0.154
	Gradual	1.01	0.92 to 1.12	0.751
Non-FHCI	Immediate	3.82	2.96 to 4.92	<0.001
	Gradual	0.85	0.81 to 0.90	<0.001

Non-FHCI = not included in the Free Health Care Initiative.

**Table 5:** Estimated recurrent cost of the intervention.

Title 1	Title 2	Title 3
NEMS Generator Fuel	7	561
Ambulance Fuel	439	35,567
Ambulance Maintenance	336	27,207
GPS	23	1903
Mobile Phones Air-time ^1^	32	2626
Medical Material and PPEs ^2^	94	7574
Insurance	16	1296
Paramedics salary ^3^	1406	113,850
OC ^4^ operators’ salary	94	7590
Drivers’ salary	1178	95,400
Total	3624	293,574

Costs are expressed in US dollars, with an exchange rate of 1 USD = 10235 Sierra Leone Leones; ^1^ Mobile phones were provided to all the ambulances (*n* = 81); ^2^ Personal Protective Equipment; ^3^ Salary of personnel includes health insurance and involves 450 paramedics, 450 ambulance drivers, and 30 OC operators; ^4^ Operation Center.

## Data Availability

Restrictions apply to the availability of these data. Data were obtained from the National Referral Coordinators Database and are available from the authors with the permission of the Ministry of Health and Sanitation of Sierra Leone.

## Supplementary Materials

The following are available online at https://www.mdpi.com/article/10.3390/ijerph18189546/s1, Table S1: Classification of NEMS obstetric emergencies triaged as “red” codes.
