# Supplementary material for: Improving Access to Healthcare in Sierra Leone: The Role of the Newly Developed National Emergency Medical Service
Source: Int J Environ Res Public Health. 2021 Sep 10;18(18):9546. doi: 10.3390/ijerph18189546 (PMC8472563; doi:10.3390/ijerph18189546)
Supplement: Supplementary file 1 [file ijerph-18-09546-s001.zip › ijerph-1360253-supplementary.pdf]

Supplementary Material

Table S1. Classification of NEMS obstetric emergencies triaged as "Red" codes.

Type of emergency
Pregnancy-related
Antepartum hemorrhage
Hypertensive disorders
Anemia
Abortion
PROM*
Ectopic pregnancy
Delivery-related
Obstructed labour
Postpartum hemorrhage
Fetal distress
Other
Severe malaria
Respiratory distress
Trauma
Severe diarrhea and dehydration
Sepsis

* PROM: premature rupture of membranes.
